# When a Small Amount of Comonomer Is Enough: Tailoring the Critical Solution Temperature of LCST-Type Thermoresponsive Random Copolymers by PEG Methyl Ether Methacrylate with 1100 g/mol Molecular Weight

**DOI:** 10.3390/ma18020372

**Published:** 2025-01-15

**Authors:** György Kasza, Bence Sármezey, Dóra Fecske, Klára Verebélyi, Béla Iván

**Affiliations:** 1 Polymer Chemistry and Physics Research Group, Institute of Materials and Environmental Chemistry, HUN-REN Research Centre for Natural Sciences, Magyar Tudósok Körútja 2, H-1117 Budapest, Hungary; sarmezey.bence@ttk.hu (B.S.); fecske.dora@ttk.hu (D.F.); verebelyi.klara@ttk.hu (K.V.); 2 Hevesy György Doctoral School of Chemistry, ELTE Eötvös Loránd University, Pázmány Péter sétány 1/A, H-1117 Budapest, Hungary

**Keywords:** thermoresponsive polymers, statistical copolymers, critical solution temperature, CST, cloud point, clearing point, PEG-methacrylate, mPEGMA, DEGEEA, *N*-isopropyl acrylamide, *N*-isopropyl methacrylamide

## Abstract

Tuning the critical solution temperature (CST) of thermoresponsive polymers is essential to exploit their immense potential in various applications. In the present study, the effect of PEG-methyl ether methacrylate with a higher molecular weight of 1100 g/mol (mPEGMA_1100_) as a comonomer was investigated for its suitability for the CST adjustment of LCST-type polymers. Accordingly, a library of mPEGMA_1100_-based copolymers was established with varying compositions (*X*_mPEGMA1100_) using four main comonomers, namely di(ethylene glycol) ethyl ether acrylate, *N*-isopropyl acrylamide and methacrylamide, and mPEGMA_300_, with different CST values (cloud points, *T_CP_*, and clearing points, *T_CL_*, by turbidimetry). It was found that less than 20 mol% of the mPEGMA_1100_ in the copolymers is practically sufficient for tuning the CST in the entire measurable temperature range, i.e., up to 100 °C, regardless of the CST of the homopolymer of the main comonomer (CST_0_). Moreover, a predictive asymptotic model was developed based on the measured CST values, which strikingly revealed that the CSTs of mPEGMA_1100_-containing copolymers depend only on the two main parameters of these copolymers, *X*_mPEGMA1100_ and the CST of the homopolymer of the main comonomer (CST_0_), that is, *CST = f(CST_0_, X_mPEGMA1100_)*. The revealed two-parameter relationship defines a surface in 3D plotting, and it is applicable to determine the CST of copolymers in advance for a given composition or to define the suitable composition for a required CST value. These unprecedented results on the dependence of CSTs on two major well-defined parameters enable to design a variety of novel macromolecular structures with tailored thermoresponsive properties.

## 1. Introduction

Undisputedly, thermoresponsive polymers belong to one of the most intensively investigated fields of material science and technology due to their high potential for various applications [1,2,3,4,5,6,7,8]. The thermoresponsivity of polymer solutions means either lower-critical-solution-temperature (LCST)- or upper-critical-solution-temperature (UCST)-type behavior, where LCST and UCST refer to the temperatures at the minimum or maximum in the critical solution temperature (CST) versus polymer/solvent fraction phase diagrams, respectively. To date, numerous polymers have been demonstrated to possess LCST-type thermoresponsive behavior (see e.g., Refs. [1,2,3,4,5,6,7,8,9,10,11,12,13,14,15,16,17,18,19,20,21,22] and references therein). These include various *N*-alkyl (meth)acrylamides, poly(2-alkyl-2-oxazoline)s, poly(methyl vinyl ether), poly(ethylene glycol)s (PEG) and its polymacromonomers, and poly(*N*,*N*-dimethylaminoethyl methacrylate), among others. It is of paramount importance to be able to adjust the CST value of such polymers. The CST can be tuned in several ways, including forming interpolymer complexes and designing new monomers based on structural analogies [23,24,25,26]. However, the most convenient approach is the copolymerization of selected comonomers.

Oligo(ethylene glycol) and poly(ethylene glycol) (PEG)-based macromonomers are well-suited for CST regulation purposes. It has been demonstrated that polymers constructed from these PEG macromonomers exhibit thermoresponsive behavior [14,15,16,17,18,27,28,29,30,31,32,33,34,35,36,37,38,39,40,41,42,43,44]. The number of ethylene glycol repeat units in the side chains, ranging from two to nine, regulates the extent of hydrogen bonding with water, which allows for control of the CST value to the desired temperature by selecting the appropriate length of the PEG monomer. On top of this, PEGs have many advantageous properties, such as water solubility and biocompatibility; therefore, it is not surprising that the PEG-based monomers have been extensively utilized and investigated in various copolymers. For instance, PEG macromonomers were copolymerized with hydrophobic monomers, such as (meth)acrylates [29,31,40], styrene [45], or glycidyl methacrylate [46], as well as with hydrophilic comonomers, including 2-hydroxyethyl methacrylate [37] and methacrylic acid [15,16], among others. Various poly(ethylene glycol) (meth)acrylates (PEG(M)As) were also copolymerized with monomers the homopolymers of which exhibit LCST-type behavior, including NiPAAm [17,27,28,44], *N*,*N*-diethylacrylamide (DEAAm) [47], DMAEMA [18,33,34], and *N*-vinylcaprolactam [39]. In certain cases, a correlation was reported between the composition of the copolymers and their CST [16,32,36,40,48]. A nonlinear composition dependence of the cloud point temperature (*T*_CP_) was observed for copolymers of poly(ethylene glycol) methacrylates (PEGMAs) with methacrylic acid (MMA) [16], as well as for PEGMA and specific alkyl methacrylates [40]. Other studies have reported a linear *T*_CP_-composition relationship for various comonomer pairs, including di(ethylene glycol) methyl ether methacrylate (DEGMEMA) and PEGMA with a molecular weight of 300 g/mol [48] and 475 g/mol [32], as well as di(ethylene glycol) ethyl ether acrylate (DEGEEA) and *N*,*N*-dimethylacrylamide (DMAAm) [36].

Despite the extensive research conducted with several PEGMAs with low molecular weights (e.g., DEGMEMA, TriEGMEMA, mPEGMA_300_, and mPEGMA_500_), surprisingly, limited research has explored the applications of higher-molecular-weight PEG monomers, such as the commercially available mPEGMA with a molecular weight of 1100 g/mol (mPEGMA_1100_) as comonomers in thermoresponsive copolymers. The copolymerization of mPEGMA_1100_ with DEGMEMA in the composition range of 1 to 10 mol% of mPEGMA_1100_ resulted in an observable increase in the CST value up to approximately 61 °C [49]. As anticipated, copolymers of mPEGMA_1100_ and NiPAAm have also been investigated, but the reported results are somewhat contradictory. Kim and coworkers synthesized a copolymer series of these comonomers, and the *T*_CP_ was 57.3 °C when the mPEGMA_1100_ content in the copolymer was 2.5 mol% [17]. However, the *T*_CP_ could not be determined for higher mPEGMA_1100_ contents. Nevertheless, they subsequently reported that the P(NiPAAm-*co*-mPEGMA_1100_) with a 1 mol% PEGMA content exhibited a *T*_CP_ of approximately 58 °C [50]. These contradictions can be attributed to the measurement method of the CST, which was determined by using an optical microscope, and the percentage of light blockage expressed the transition effect. In contrast, an increasing CST up to approximately 95 °C was reported in the case of a star-like copolymer containing NiPAAm and mPEGMA_1100_ with a hyperbranched polyester core and 0.476 molar ratio of [PEGMA]/[NiPAAm] comonomers [51].

The above studies indicate that the CST of thermoresponsive copolymers can be controlled based on the composition by utilizing mPEGMA_1100_ in a maximum of 10-15 mol% of this comonomer. However, there has not been any systematic investigation published on the effect of the mPEGMA_1100_ comonomer on the CST of thermoresponsive copolymers with varying compositions to the best of our knowledge. According to our concept, by understanding the effect of the mPEGMA_1100_ content on the CST of its copolymers within this compositional range, copolymers with predetermined thermoresponsive behavior can be designed and obtained. Herein, to ascertain the applicability of mPEGMA_1100_ for such purposes and to reveal the effect of the composition on the thermoresponsive behavior, a library of copolymers was prepared using mPEGMA_1100_ with four selected comonomers, namely DEGEEA, NiPAAm, NiPMAAm, and mPEGMA_300_, for which the homopolymers possess quite different CSTs. Subsequently, the performed correlation analysis on the observed transition temperatures and the structural parameters of the copolymers revealed a relationship between the CST and two parameters, i.e., the composition of the copolymers and the CST value of the homopolymer of the main comonomer. This correlation enables the design of thermoresponsive copolymers possessing the desired critical solution temperatures across the measurable temperature range.

## 2. Materials and Methods

### 2.1. Materials

Di(ethylene glycol) ethyl ether acrylate (DEGEEA, technical grade, from Sigma-Aldrich, Steinheim, Germany) and poly(ethylene glycol) methyl ether methacrylates (mPEGMA_300_ and mPEGMA_1100_, all technical grade, from Sigma-Aldrich, Steinheim, Germany) were passed through a column packed with basic alumina to remove inhibitors, and the purified macromonomers were stored until use at 4 °C under a nitrogen atmosphere. *N*-isopropyl acrylamide (NiPAAm, 97%, Sigma-Aldrich, Steinheim, Germany) and *N*-isopropyl methacrylamide (NiPMAAm, 97%, Sigma-Aldrich, Steinheim, Germany) were recrystallized from *n*-hexane twice. Azobisisobutyronitrile (AIBN, 98%, Sigma-Aldrich, Steinheim, Germany) was purified by recrystallization from methanol before use. Tetrahydrofuran (THF, VWR International Ltd., Debrecen, Hungary) was refluxed over sodium benzophenone for one hour and distilled at 66 °C. *n*-Hexane (from VWR International Ltd., Debrecen, Hungary) was used as received.

### 2.2. Synthesis of Homopolymers and Copolymers

The homopolymers and mPEGMA_1100_-based copolymers were prepared by free radical copolymerization. Briefly, the required amount of purified comonomers was measured into a sealable vial, and then, the sufficient stock solution of the AIBN initiator (100 mg/mL AIBN in THF) was added, and the volume was adjusted to 10 mL with THF. The vial was sealed, and the reaction mixture was deoxygenated by bubbling with nitrogen for half an hour. The vials were tempered to 65 °C while stirring for 12 h. The products were obtained via precipitation into a large excess of *n*-hexane and then were dried in a vacuum at 65 °C to a constant weight. In all cases, the monomer/initiator ratio was 100:1, and the comonomer ratio was systematically varied to study the effect of the composition on the thermoresponsive behavior of the prepared copolymers. The measured weights of all reactants in these polymerizations with the obtained yields are listed in Table S1 in the Supporting Information.

### 2.3. Characterization Methods

#### 2.3.1. Gel Permeation Chromatography (GPC)

The synthesized polymers were characterized using a GPC equipped with a Waters 515 HPLC pump (Milford, MA, USA), Waters 717 Plus autosampler (Milford, MA, USA), Agilent 1260 Infinity differential refractometric detector (Santa Clara, CA, USA), Waters Styragel HR1 and HR4 columns connected in series and thermostated at 35 °C. The eluent was THF with a flow rate of 0.3 mL/min. The chromatograms were evaluated through conventional calibration with linear polystyrene standards (PSS Polymer Standards Service GmbH, Mainz, Germany).

#### 2.3.2. Proton Nuclear Magnetic Resonance (^1^H NMR)

^1^H NMR spectroscopy measurements were performed on a Varian iNOVA 500 spectrometer operating (Santa Clara, CA, USA) at a 500 MHz ^1^H frequency in D_2_O at room temperature.

#### 2.3.3. Turbidimetric Measurements

Transmittance versus temperature curves, to obtain the critical solution temperatures (CST or *T*_C_), were measured using a UV-Vis spectrophotometer (Jasco V-650, JASCO Corporation, Tokyo, Japan) equipped with a Jasco MCB-100 mini circulating bath (JASCO Corporation, Tokyo, Japan) and a Peltier thermostat. Deionized water was used as a reference and solvent. The transmittance of the polymer solutions (1 mg/mL) was recorded at 480 nm during the heating and then the cooling cycle (heating/cooling rate of 0.2 °C/min with 5 min waiting time for equilibration between temperature-changing steps). The CST is defined as the temperature at the inflection point of the transmittance–temperature curves, i.e., the so-called cloud point temperature (*T*_CP_) for heating and the clearing point temperature (*T*_CL_) for cooling [52,53].

## 3. Results and Discussion

**Scheme 1:**
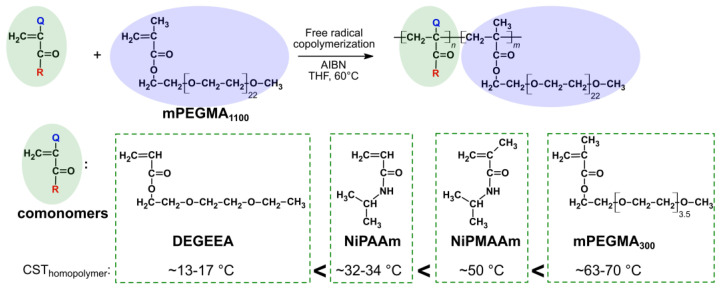
Synthesis of mPEGMA_1100_-based thermoresponsive copolymers via free radical copolymerization using different comonomers, such as di(ethylene glycol) ethyl ether acrylate (DEGEEA), *N*-isopropyl acrylamide (NiPAAm), *N*-isopropyl methacrylamide (NiPMAAm), and PEG-methyl ether methacrylate with 300 g/mol molecular weight (mPEGMA_300_).

Our study aimed at exploring how a higher-molecular-weight mPEGMA macromonomer (1100 g/mol) could impact the critical solution temperature (CST) of thermoresponsive polymers via copolymerization. Accordingly, a copolymer library was produced using mPEGMA_1100_, which was copolymerized with different main comonomers, the homopolymers of which possess distinct CST values in a wide temperature range, and different polymerizable groups, specifically DEGEEA, NiPAAm, NiPMAAm, and mPEGMA_300_. Each set of copolymers was prepared by free radical copolymerization with four different compositions of up to 15 mol% mPEGMA_1100_ contents. The synthetic approach used is presented in Scheme 1.

The GPC chromatograms, the molecular weight distribution curves, and detailed average molecular weight and dispersity data of the investigated copolymers are presented in Figures S1–S4 and Table S2, respectively. As observed, the copolymers in the different series were obtained in the same molecular weight range with dispersity values between approximately 1.7 and 2.7, typical for free radical polymerizations. It is important to note that concerning the dependence of the critical solution temperature on the molecular weight, previous studies reported a decrease, increase, and independence as well [22,54,55,56,57,58]. From this point of view, free radical polymerization may be advantageous due to the relatively broad molecular weight distribution of the resulting copolymers, so that the effect of molecular weight is expected to become negligible. In the case of NiPAAm- and NiPMAAm-based copolymers, the GPC chromatograms indicate a bimodal molecular weight distribution, which can be attributed to the enhanced occurrence of recombination as a termination reaction in radical polymerizations. However, it must be emphasized that this does not affect the composition of the copolymers, i.e., the branching density of the obtained mPEGMA_1100_-based copolymers.

The composition of the prepared copolymers, i.e., the ratio of the comonomers incorporated into the copolymers, was determined by ^1^H NMR spectroscopy. The recorded spectra of the products are displayed in Figures S5–S8. The molar fraction of the comonomers in the feed and the obtained copolymers, i.e., the theoretical and determined compositions, are listed in Table S2 and displayed in Figure S9. The resulting composition exhibited slight differences from the feed, with a higher incorporation of mPEGMA_1100_ relative to the feed, except for P(mPEGMA_300_-*co*-mPEGMA_1100_), where the polymerizable functional group is the same and the obtained composition closely matches the feed. The observed discrepancy can be attributed to the differences in reactivity ratios of the comonomers. The high reactivity of mPEGMA_1100_ was demonstrated via its copolymerization with di(ethylene glycol) methyl ether methacrylate; however, the copolymerization ratios of this comonomer are not known for other comonomers. On the one hand, considering that the product of the copolymerization ratios in the radical copolymerization of acrylamides and methacrylates is close to one, e.g., in the case of NIPAAm and glycidyl methacrylate (r_1_ = 0.39 and r_2_ = 2.69) [59], the formation of random P(NiPAAm-*co*-mPEGMA_1100_) copolymers is expected. On the other hand, the higher reactivity of mPEGMA than that of the acrylates and methacrylamides in various comonomer pairs was also demonstrated. For instance, the reactivity ratios of tert-butyl 2-((2-bromopropanoyloxy)methyl)acrylate and mPEGMA500 are 0.32 and 1.44 [60], while for butyl acrylate and methyl methacrylate, they are 0.32 and 1.94 [61]. Nevertheless, the reactivity ratios have also been reported in the case of methacrylamide and methacrylate comonomer pairs. For example, the reactivity ratios for the pairs *N*-phenylmethacrylamide with glycidyl methacrylate and 2-hydroxyethyl methacrylate with methacrylamide were 2.44–0.23 [62] and 1.53–0.3 [63], respectively. Since these reactivity ratios for all the mentioned comonomer pairs are not greater than 1.0 in either case, and because the products of the reactivity ratios are also less than 1.0 (r_1_r_2_ < 1, r_1_ < 1 and r_2_ > 1), it can be concluded that the sequence of the produced mPEGMA_1100_-based copolymers is statistical in the resulting copolymers.

**Figure 1:**
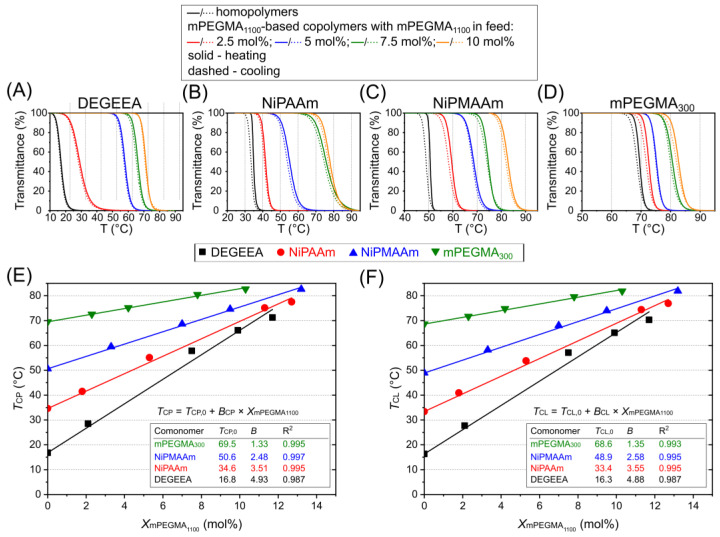
The transmittance–temperature curves of various homopolymers and the mPEGMA_1100_-containing copolymers (**A**–**D**), and the obtained cloud point (*T*_CP_) and the clearing point (*T*_CL_) temperatures as a function of the molar fraction of the mPEGMA_1100_ comonomer (**E**,**F**).

The thermoresponsive behavior of the copolymers was investigated by turbidimetric measurements. The obtained transmittance–temperature curves for the prepared polymers are presented in Figure 1A–D. The first derivatives of these curves are displayed in Figures S10–S13. The minima of the derivatives provide the *T*_CP_ and *T*_CL_ values with the extent of the hysteresis listed in Table S2.

As can be seen in Figure 1A–D, the transmittance–temperature curves of the heating and cooling cycles show reversible thermoresponsive precipitation–dissolution transitions for all the mPEGMA_1100_-containing copolymers. As shown in these Figures, the composition of the copolymers has a major influence on the thermoresponsive behavior of all the copolymers, as evidenced by the fact that the transmittance–temperature curves are shifted to higher temperatures by increasing the mPEGMA_1100_ comonomer content. Moreover, all the copolymers with even the highest mPEGMA_1100_ content exhibit a thermoresponsive transition within the measurable temperature range, approximately between 70 and 85 °C, regardless of the CST values of the pure homopolymers. Consequently, to investigate the molar effect of the mPEGMA_1100_ on the CST, the *T*_CP_ and the *T*_CL_ values were plotted as a function of the molar fraction of mPEGMA_1100_ (*X*_mPEGMA1100_) determined by ^1^H NMR spectroscopy (see Figure 1E,F for *T*_CP_ and *T*_CL_, respectively). The *T*_CP_ and *T*_CL_ of all the copolymers exhibit a linear dependence on the mPEGMA_1100_ content within the investigated composition range.

Thus, a linear relationship can be defined for both the cloud points and clearing points according to the following equations:

(1) $$T_{CP}=T_{CP,0}+B_{CP}\times X_{mPEGMA1100}$$

(2) $$T_{CL}=T_{CL,0}+B_{CL}\times X_{mPEGMA1100}$$

where *T*_CP,0_ and *T*_CL,0_ are the cloud point and clearing point temperatures of the pure homopolymer, respectively, the *B*_CP_ and the *B*_CL_ are the corresponding slopes, and X_mPEGMA1100_ is the molar fraction of the mPEGMA_1100_ comonomer in the copolymer.

**Figure 2:**
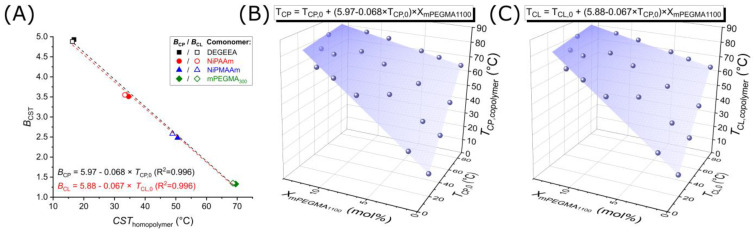
The slope (*B*_CST_) of the fitted lines of the CST vs. *X*_mPEGMA1100_ data as a function of the CST of the homopolymers (**A**, filled symbols for *T*_CP_ and open symbols for *T*_CL_) and a three-dimensional plot of the *T*_CP_ (**B**) and *T*_CL_ (**C**) values of the mPEGMA_1100_-based copolymers as a function of the composition (*X*_mPEGMA1100_) and the *T*_CP_ and *T*_CL_ of the homopolymers.

It is noteworthy to highlight that the *B* value decreases as the CST value of the homopolymer increases, suggesting a potential correlation between these parameters. Therefore, the determined slopes were plotted as a function of the CST values of the pure homopolymers (see Figure 2A). Unexpectedly, the obtained data could be fitted well by a linear function (R^2^ is 0.996 for both *B*_CP_ and *B*_CL_), indicating that the slopes of the *T*_CP_ and *T*_CL_ versus *X*_mPEGMA1100_ plots in Figure 1E,F exclusively depend on the CST of the homopolymers but are independent of the type of the polymerizable group of the main comonomer. Thus, knowing the CST values of the homopolymers, the slope (*B*) of the linear function describing the relationship between the CST values of the copolymers and the composition in the studied range can be estimated using the following equations:

(3) $$B_{CP}=5.97-0.068\times T_{CP,0}$$

(4) $$B_{CL}=5.88-{0.067\times T}_{CL,0}$$

Consequently, the slopes can be substituted by these linear equations in Equations (1) and (2), respectively, and the following correlations are obtained for the critical solution temperatures, i.e., *T*_CP_ and *T*_CL_:

(5) $$T_{CP}=T_{CP,0}+(5.97-0.068\times T_{CP,0})\times X_{mPEGMA1100}$$

(6) $$T_{CL}=T_{CL,0}+(5.88-0.067\times T_{CL,0})\times X_{mPEGMA1100}$$

These equations mean that the *T*_CP_ and *T*_CL_ of the thermoresponsive copolymers containing mPEGMA_1100_ comonomer depend only on two major structural parameters, which are the molar fraction of the mPEGMA_1100_ comonomer in the thermoresponsive copolymers and the CST value of the homopolymer of the main comonomer. This dependence can be displayed in a three-dimensional plot, as presented for *T*_CP_ and *T*_CL_ in Figure 2B,C, respectively. These 3D graphs are shown rotating in Figure S14 in a separate file in the Supporting Information to provide a better visual overview. The resulting slightly twisted planar surfaces, described by Equations (5) and (6), allow tuning of the CST values of thermoresponsive copolymers with the mPEGMA_1100_ comonomer by a good estimation.

However, as can be seen in Figure 1E,F, the lines fitted to the *CST*-*X*_mPEGMA1100_ data intersect approximately in the range of 11–15 mol% mPEGMA_1100_ and are moving away from each other, although they should be intended for a given temperature value as the comonomer content increases, namely the CST value of the pure poly(mPEGMA_1100_) homopolymer. The fact that these fitted lines intersect means that this model defined by Equations (5) and (6) (hereafter referred to as the linear–linear model) is valid only below a 12–15 mol% mPEGMA_1100_ content. Additionally, in the case of the homopolymers with lower CSTs, the accuracy of the linear–linear model decreases, which can be attributed to the difference between the calculated and the fitted slopes (*B*). Despite the linear fit describing the *B*-CST relationship being reasonably good, this approximation is flawed because the *B* should only be equal to zero when the CST of the homopolymer corresponds to the P(mPEGMA_1100_) homopolymer. However, solving Equation (3) for *B* = 0 gives a CST value of approximately 88 °C, which is lower than the CST of the P(mPEGMA_500_) homopolymer (~95 °C) reported in the literature [64].

**Figure 3:**
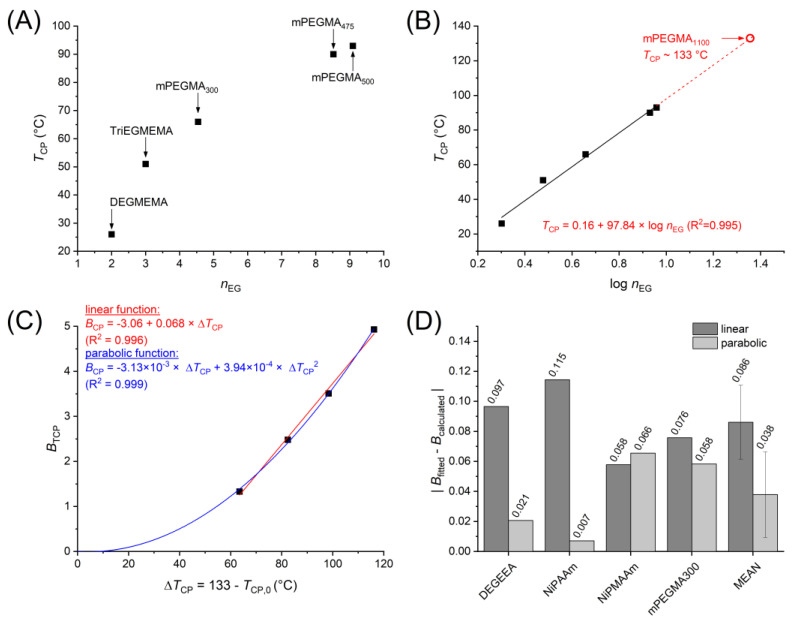
The cloud point temperature (*T*_CP_) as a function of the number of ethylene glycol (*n*_EG_) repeat units of the PEG-methacrylate homopolymers (**A**, data from Refs. [32,64,65,66]), and as a function of the logarithm of *n*_EG_ (**B**). The slope (*B*_CP_) of the fitted lines of the *T*_CP_ vs. *X*_mPEGMA1100_ data as a function of the difference between the virtual *T*_CP_ of P(mPEGMA_1100_) and the homopolymers of the main comonomers (Δ*T*_CP_) (**C**) and the absolute value of the difference between the measured and calculated slopes using linear and parabolic determination method (**D**).

To evolve the linear–linear estimation model, it is necessary to ascertain the CST of the P(mPEGMA_1100_) homopolymer. It is well-known that the CST of the polyPEGMA homopolymers increases with an increasing number of ethylene glycol units in the side chain (see Figure 3A, the data are adapted from Refs. [32,64,65,66]). However, when the CST values of the P(mPEGMA) homopolymers are plotted as a function of the logarithm of the number of ethylene glycol units (see Figure 3B), the resulting graph reveals an unexpected linear correlation with a sufficiently high R-squared value (R^2^ = 0.995). The equation of the fitted linear function was then employed to estimate the CST of the P(mPEGMA_1100_) homopolymer, with the extrapolation suggesting that the virtual CST (or hypothetical CST) of the P(mPEGMA_1100_) homopolymer is approximately 133 °C.

Subsequently, the *B* slope values of the fitted lines in the *T*_CP_ versus *X*_mPEGMA1100_ graphs were plotted as a function of the difference between the estimated *T*_CP_ of the P(mPEGMA_1100_) and the *T*_CP_ of the corresponding homopolymers (Δ*T*_CP_ = 133 − *T*_CP,0_). As can be seen in Figure 3C, the data can be accurately fitted with a parabolic function (R^2^ = 0.999) starting from the origin, i.e., where the given CST value is equal to the CST value of P(mPEGMA_1100_), then the slope is zero in the *B*_CP_ versus Δ*T*_CP_ plot. This parabolic function is described by the following equation:

(7) $$B_{CP}=-3.13\times 10^{-3}\times {∆T}_{CP}+3.94\times 10^{-4}\times {∆T}_{CP}^{2}$$

A comparison of the absolute difference between the slope *B* values determined by the linear and parabolic functions (Equations (3) and (7)) and the slope of the original fitted lines in the *T*_CP_ versus *X_mPEGMA110_* plot (see Figure 3D) demonstrates that the slope can be estimated with greater accuracy using the parabolic function. This is evidenced by a reduction in the absolute residual values, which are practically less than 0.07 for all the copolymers, and the average absolute residual of the parabolic function is approximately half of the linear model, namely 0.038 and 0.086, respectively. On the one hand, the high R-squared value of this parabolic function also indicates that the estimation of the virtual CST of the P(mPEGMA_1100_) homopolymer may be sufficiently accurate despite its hypothetical nature. On the other hand, the linear equation describing the *B* slope in the linear–linear estimation model can be replaced by the parabolic function, as follows:

(8) $$T_{CP}=T_{CP,0}+(-3.13\times 10^{-3}\times {∆T}_{CP}+3.94\times 10^{-4}\times {{∆T}_{CP}}^{2})\times X_{mPEGMA1100}$$

The Equation (8), which can be represented as a three-dimensional parabolic surface (see the rotating plot in Figure S15 in the Supporting Information), serves as an accurate model (being referred to as the linear-parabolic model) for the determination of the *T*_CP_ of the statistical copolymers containing the mPEGMA_1100_ comonomer. Similar to the linear–linear model, this model still depends only on two independent variables, namely the *T*_CP_ of the homopolymer and the composition (X_mPEGMA1100_). Even though the accuracy of the linear–parabolic model is greater than that of the linear–linear model, the CST of the copolymers still depends linearly on the composition, so that the linear–parabolic estimation model is still applicable up to approximately a 10–15 mol% mPEGMA_1100_ comonomer content.

**Figure 4:**
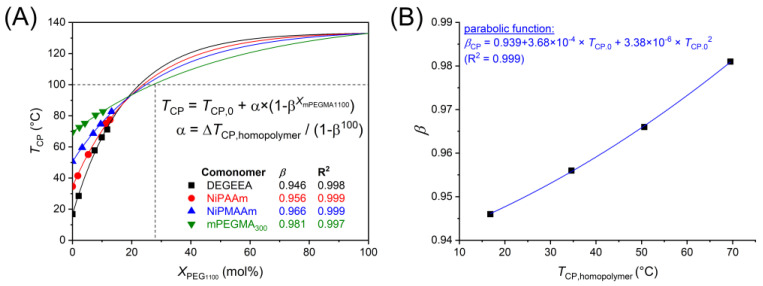
The cloud point temperature (*T*_CP_) of the mPEGMA_1100_-based copolymers as a function of the molar fraction of the mPEGMA_1100_ comonomer in the whole composition range (**A**), and the dependence of the *b* parameter of the fitted asymptotic function on the *T*_CP_ of the homopolymers (**B**).

To extend the applicability of the estimation model to the entire measurable range with enhanced accuracy, the available *T*_CP_-*X* data were plotted over the whole composition scale with the assumed *T*_CP_ value of P(mPEGMA_1100_) homopolymer as *T*_CP,100_ (see Figure 4A). These datasets were then fitted with a shifted asymptotic function, described by the following equation:

(9) $$T_{CP}=T_{CP,0}+\alpha \times (1-\beta^{X_{mPEGMA1100}})$$

To ensure that the fitted curves intersect each other at the virtual CST of the pure P(mPEGMA_1100_) homopolymer (133 °C), the *α* coefficient was expressed as follows and substituted in Equation (9):

(10) $$\alpha =\frac{T_{CP,100}-T_{CP,0}}{\left(1-\beta^{100}\right)}=\frac{{∆T}_{CP}}{\left(1-\beta^{100}\right)}$$

(11) $$T_{CP}=T_{CP,0}+\frac{{∆T}_{CP}}{\left(1-\beta^{100}\right)}\times (1-\beta^{X_{mPEGMA1100}})$$

As displayed in Figure 4A, the function described by Equation (11) accurately fits the data sets with R-squared values above 0.997 in all copolymer cases. To create a reliable estimation model for the CST of the mPEGMA_1100_-based copolymers, the relationship between the *β* coefficient and a measurable parameter, specifically the *T*_CP_ of the homopolymer of the main comonomer, was explored. As shown in Figure 4B, the *β* versus *T*_CP,0_ data are well-fitted by a parabolic function (R^2^ = 0.999), indicating that the *β* coefficient of the asymptotic function can be determined using the *T*_CP_ of the main comonomer’s homopolymer and its corresponding coefficients, as follows:

(12) $$\beta =0.939+3.68\times 10^{-4}\times T_{CP,0}+3.38\times 10^{-6}\times {T_{CP,0}}^{2}$$

**Figure 5:**
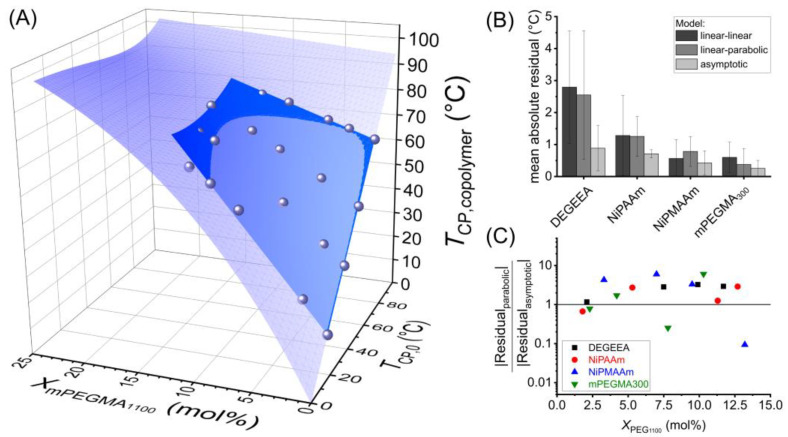
The three-dimensional plot of the *T*_CP_ values of mPEGMA_1100_-based copolymers as a function of the composition (*X*_mPEGMA1100_) and the *T*_CP_ of the homopolymer of the main comonomer with the surfaces representing the asymptotic (light blue) and linear–parabolic (blue) models (**A**). The mean absolute residuals of the mPEGMA_1100_-based copolymers obtained by the different estimating models (**B**) and the ratio of the absolute residual values of the linear–parabolic and the asymptotic models as a function of the composition (**C**).

Then, the *β* coefficient in Equation (11) expressed via Equation (12) results in a two-parametric surface function, referred to as the asymptotic model, presented in Figure 5A. For a better view, this surface is shown rotating in Figure S16 in the Supporting Information. The asymptotic model is applicable across the entire measurable temperature range compared to the linear-based models, which overestimate the measured *T*_CP_ values above the 10–15 mol% comonomer range with a significant error. Although the system of the equations that describes this model is quite complex, the *T*_CP_ value of the copolymers containing mPEGMA_1100_ remains dependent only on two parameters as independent variables, namely the *T*_CP_ value of the homopolymer of the main comonomer and the composition, i.e., the mPEGMA_1100_ content (*X*_mPEGMA1100_) of the copolymers. As concluded, the CST of a specified homopolymer can be adjusted by copolymerization with mPEGMA_1100_ as a comonomer up to 20 mol% over the entire available range from the CST of the homopolymer up to 100 °C.

The accuracy of the asymptotic–parabolic model has been examined and compared to that of the linear-based models through a residual analysis. For all copolymers, the obtained *T*_CP_ values as a function of the composition with the originally fitted functions and all estimation models and their residual plots are shown in Figures S17–S20, while the measured and the calculated *T*_CP_ values with their differences are summarized in Table S3. The mean absolute residuals, i.e., the mean of the absolute values of the difference between the observed and the calculated *T*_CP_ values (see Figure 5B), reveal that the average accuracy of all models increases with an increasing *T*_CP_ of the homopolymers. However, the most accurate model is the asymptotic model, which estimates the *T*_CP_ of the copolymer with an average precision of 1 °C. Comparing the models in terms of composition is possible by examining the ratio of the absolute value of the residuals of the linear–parabolic and the asymptotic–parabolic models in the case of the different mPEGMA_1100_ comonomer contents. As shown in Figure 5C, the linear–parabolic model shows higher accuracy in instances where the mPEGMA_1100_ comonomer content is less than 2.5 mol%. Above this comonomer threshold, the estimated temperatures with low residuals are a consequence of the inherent characteristics of linear regression. However, based on these results, it should be highlighted that the accuracy of the asymptotic model is 2–8 times better than the linear–parabolic model over the entire composition range. It can be concluded that the cloud point temperature of the mPEGMA_1100_-containing copolymers can be adjusted by the composition with an accuracy of 1–1.5 °C. Furthermore, the required molar fraction of the mPEGMA_1100_ comonomer in thermoresponsive copolymers with a predetermined CST value can be well-designed in the range of 1–20 mol%.

The transition from coil to globule, involving precipitation–dissolution during heating–cooling cycles, represents two distinct processes. Since the clearing temperature values of individual mPEGMA homopolymers are unknown in several cases, it is not possible to directly estimate the *T*_CL_ values of the copolymers using linear–parabolic and asymptotic–parabolic models. However, the average of all determined hysteresis values, i.e., the difference between the *T*_CP_ and *T*_CL_ values, is 0.9 ± 0.4 °C. Therefore, it can be assumed that the *T*_CL_ value of the copolymer is lower than that of the estimated *T*_CP_ value, and the difference is within 2 °C.

It is important to note that the CST of the homopolymers and copolymers is influenced by more parameters than just the composition. Reportedly, besides the above-mentioned molecular weight effect, the dispersity and chain end functionality, which originates from the initiation/polymerization process or post-modification, also affect the CST value [19,22,55,67,68]. Therefore, the determination of the CST of the homopolymer with the correct molecular weight and end group functionality produced using a chosen polymerization method is recommended in order to avoid the potential errors resulting from these effects. In addition to the structural parameters, the determination method and its parameters affect the CST value as well. Furthermore, the measurement methods and conditions, including the concentration, heating/cooling rate, equilibration time, and even wavelength, significantly impact the obtained CST values [52,53,69]. The estimation models proposed herein were developed for use in diluted aqueous solutions of statistical copolymers (1 g/L polymer concentration) with a sufficiently slow measurement corresponding to a heating/cooling rate of 0.2 °C/min with an equilibration time of 5 min in line with the well-established proposal for *T_CP_* and *T_CL_* determination to obtain close-to-equilibrium conditions at each temperature [52,53]. It is important to note that deviations from these conditions may result in some discrepancy between the estimated and the actual measured CST values.

## 4. Conclusions

In the course of the present study, it has been found by us that the mPEGMA comonomer with a relatively high molecular weight of 1100 g/mol (mPEGMA_1100_) is readily employed for adjusting the critical solution temperature (CST, i.e., the cloud point, *T*_CP_, and clearing point, *T*_CL_, determined by turbidimetry) via copolymerization with comonomers, the homopolymers of which possess LCST-type behavior, such as mPEGMA300, DEGEEA, NiPAAm, and NiPMAAm. As observed, approximately up to about 20% of the molar fraction of the mPEGMA_1100_ comonomer is practically sufficient for tuning the CST in the measurable temperature range. In this composition region, both the *T*_CP_ and *T*_CL_ increase with an increasing mPEGMA_1100_ content, which can be well approximated with straight lines starting from the cloud points of the corresponding homopolymers in the CST versus mPEGMA_1100_ content plots. From such graphs, it is evident that the slopes (*B*_CP_ and *B*_CL_) of the obtained linear relationships decrease with an increasing *T*_CP,0_ and *T*_CL,0_ of the homopolymers. Surprisingly, these slopes as a function of the transition temperatures of the homopolymers fall on straight lines as well. Nevertheless, additional correlation analysis has revealed that using a hypothetical estimation of the virtual *T*_CP_ of the P(mPEGMA_1100_) homopolymer, a more accurate nonlinear asymptotic function-based empirical estimation model can be established. The obtained correlation based on this approach strikingly indicates that the *T*_CP_ of the copolymers depends only on two major parameters as variables, namely the copolymer composition (*X*_mPEGMA1100_) and the *T*_CP_ value of the homopolymer of the main comonomer as shown by Equations (11) and (12). On the basis of these equations, 3D plots can be generated, and plotting the data points in the corresponding plots also clearly show conclusive evidence for the validity of the obtained correlation between the CSTs of the mPEGMA_1100_-containing copolymers, their mPEGMA_1100_ contents, and the CSTs of the corresponding homopolymers (*T*_CP,0_ or *T*_CL,0_) as presented well in the rotating 3D plots in Figures S14–S16. Consequently, by considering the applicability limits of thermoresponsive polymers, the obtained relationships enable to predetermine, i.e., to design, the required composition of copolymers of mPEGMA_1100_ for a selected CST or the determination of the CST value for a given composition. These results are expected to contribute to the preparation and investigations of novel (co)polymers and macromolecular assemblies with required and/or tailored thermoresponsive properties for various applications ranging from biomedicine to bioconjugation, biomarkers, tissue engineering, drug encapsulation and sustained release, separation media, optical signaling, etc.

## Data Availability

The original contributions presented in this study are included in the article/Supplementary Materials. Further inquiries can be directed to the corresponding authors.

## Supplementary Materials

The following supporting information can be downloaded at: https://www.mdpi.com/article/10.3390/ma18020372/s1, Table S1. The applied feed ratios and amounts of the reactants used for the synthesis of the homopolymers and the mPEGMA_1100_-based copolymers, and the obtained yields. Table S2. The molar mPEGMA_1100_ comonomer content and the number average molecular weight, dispersity (*Đ*), cloud point (*T*_CP_) and clearing point (*T*_CL_), and the extent of hysteresis (*H* = *T*_CP_ − *T*_CL_) values of the homopolymers and the mPEGMA_1100_-based copolymers. Figure S1. The GPC chromatograms and the molecular weight distribution curves of the PDEGEEA homopolymer and P(DEGEEA-*co*-mPEGMA_1100_) copolymers. Figure S2. The GPC chromatograms and the molecular weight distribution curves of the PNiPAAm homopolymer and P(NiPAAm-*co*-mPEGMA_1100_) copolymers. Figure S3. The GPC chromatograms and the molecular weight distribution curves of the PNiPMAAm homopolymer and P(NiPMAAm-*co*-mPEGMA_1100_) copolymers. Figure S4. The GPC chromatograms and the molecular weight distribution curves of the PmPEGMA_300_ homopolymer and P(mPEGMA_300_-*co*-mPEGMA_1100_) copolymers. Figure S5. The ^1^H NMR spectra of the PDEGEEA homopolymer and P(DEGEEA-*co*-mPEGMA_1100_) copolymers (500 MHz, D_2_O). Figure S6. The ^1^H NMR spectra of the PNiPAAm homopolymer and P(NiPAAm-*co*-mPEGMA_1100_) copolymers (500 MHz, D_2_O). Figure S7. The ^1^H NMR spectra of the PNiPMAAm homopolymer and P(NiPMAAm-*co*-mPEGMA_1100_) copolymers (500 MHz, D_2_O). Figure S8. The ^1^H NMR spectra of the PmPEGMA_300_ homopolymer and P(mPEGMA_300_-*co*-mPEGMA_1100_) copolymers (500 MHz, D_2_O). Figure S9. The mPEGMA_1100_ content in the copolymers versus the molar fraction of the mPEGMA_1100_ comonomer in the feed. Figure S10. Transmittance–temperature curves (left) and their derivatives (right) during heating (solid lines) and cooling (dashed lines) of the PDEGEEA homopolymer and P(DEGEEA-*co*-mPEGMA_1100_) copolymers. Figure S11. Transmittance–temperature curves (left) and their derivatives (right) during heating (solid lines) and cooling (dashed lines) of the PNiPAAm homopolymer and P(NiPAAm-*co*-mPEGMA_1100_) copolymers. Figure S12. Transmittance–temperature curves (left) and their derivatives (right) during heating (solid lines) and cooling (dashed lines) of the PNiPMAAm homopolymer and P(NiPMAAm-*co*-mPEGMA_1100_) copolymers. Figure S13. Transmittance–temperature curves (left) and their derivatives (right) during heating (solid lines) and cooling (dashed lines) of the PmPEGMA_300_ homopolymer and P(mPEGMA_300_-*co*-mPEGMA_1100_) copolymers. Figure S14. The rotating 3D plot of the cloud point (*T*_CP_, left) and clearing point (*T*_CL_, right) as a function of the composition (*X*_mPEGMA1100_) in the mPEGMA_1100_-based copolymers and the *T*_CP_ and *T*_CL_ of the homopolymer of the main comonomer with the fitted surface of the linear–linear estimation model. Figure S15. The rotating 3D plot of the cloud point (*T*_CP_) as a function of the composition (*X*_mPEGMA1100_) in the mPEGMA_1100_-based copolymers and the *T*_CP_ of the homopolymer of the main comonomer with the fitted surface of the linear–parabolic estimation model. Figure S16. The rotating 3D plot of the cloud point (*T*_CP_) as a function of the composition (*X*_mPEGMA1100_) in the mPEGMA_1100_-based copolymers and the *T*_CP_ of the homopolymer of the main comonomer with the fitted surface of the asymptotic estimation model. Figure S17. The cloud point temperature (*T*_CP_) of the P(DEGEEA-*co*-mPEGMA_1100_) copolymers as a function of the molar fraction of the mPEGMA_1100_ comonomer with the original fitted linear and asymptotic functions and the estimated *T*_CP_ − *X*_mPEGMA1100_ relationships using different models (A). The residual plot, i.e., the difference between the measured and the fitted and the calculated *T*_CP_ values, as a function of the composition (B). Figure S18. The cloud point temperature (*T*_CP_) of the P(NiPAAm-*co*-mPEGMA_1100_) copolymers as a function of the molar fraction of the mPEGMA_1100_ comonomer with the original fitted linear and asymptotic functions and the estimated *T*_CP_ − *X*_mPEGMA1100_ relationships using different models (A). The residual plot, i.e., the difference between the measured and the fitted and the calculated *T*_CP_ values, as a function of the composition (B). Figure S19. The cloud point temperature (*T*_CP_) of the P(NiPMAAm-*co*-mPEGMA_1100_) copolymers as a function of the molar fraction of the mPEGMA_1100_ comonomer with the original fitted linear and asymptotic functions and the estimated *T*_CP_ − *X*_mPEGMA1100_ relationships using different models (A). The residual plot, i.e., the difference between the measured and the fitted and the calculated *T*_CP_ values, as a function of the composition (B). Figure S20. The cloud point temperature (*T*_CP_) of the P(mPEGMA_300_-*co*-mPEGMA_1100_) copolymers as a function of the molar fraction of the mPEGMA_1100_ comonomer with the original fitted linear and asymptotic functions and the estimated *T*_CP_ − *X*_mPEGMA1100_ relationships using different models (A). The residual plot, i.e., the difference between the measured and the fitted and the calculated *T*_CP_ values, as a function of the composition (B). Table S3. The measured and the estimated *T*_CP_ values of the mPEGMA_1100_-based copolymers were obtained via the original fitted functions and using the developed estimation models with the mean absolute residuals.
