# Supplementary material for: When a Small Amount of Comonomer Is Enough: Tailoring the Critical Solution Temperature of LCST-Type Thermoresponsive Random Copolymers by PEG Methyl Ether Methacrylate with 1100 g/mol Molecular Weight
Source: Materials (Basel). 2025 Jan 15;18(2):372. doi: 10.3390/ma18020372 (PMC11766903; doi:10.3390/ma18020372)
Supplement: Supplementary file 1 [file materials-18-00372-s001.zip › materials-3412885-supplementary.pdf]

materials

MDPI

SUPPORTING INFORMATION

When a Small Amount of Comonomer is Enough: Tailoring the Critical Solution Temperature of LCST-Type Thermoresponsive Random Copolymers by PEG Methyl Ether Methacrylate with 1100 g/mol Molecular Weight

György Kasza [1,*], Bence Sármezey [1,2], Dóra Fecske [1,2], Klára Verebélyi [1] and Béla Iván [1,*]

[1] Polymer Chemistry and Physics Research Group, Institute of Materials and Environmental Chemistry, HUN-REN Research Centre for Natural Sciences, Magyar tudósok körútja 2, H-1117 Budapest, Hungary; sarmezey.bence@ttk.hu; fecske.dora@ttk.hu; verebelyi.klara@ttk.hu

[2] Hevesy György Doctoral School of Chemistry, ELTE Eötvös Loránd University, Pázmány Péter sétány 1/A, H-1117 Budapest, Hungary

* Correspondence: kasza.gyorgy@ttk.hu (Gy.K.); ivan.bela@ttk.hu (B.I.)

Table S1. The applied feed ratios and amounts of the reactants used for the synthesis of the homopolymers and the $mPEGMA_{1100}$-based copolymers, and the obtained yields.

main comonomer	molar ratio			*m* (g)			Yield (%)
	$mPEGMA_{1100}$	main comonomer:	AIBN	main comonomer	$mPEGMA_{1100}$	AIBN	
NiPAAm	0	100	1	0.9857	0.0000	0.014	62
	2.5	97.5	1	0.7911	0.1972	0.012	86
	5	95	1	0.6549	0.3351	0.010	63
	7.5	92.5	1	0.5544	0.4369	0.009	68
	10	90	1	0.4771	0.5153	0.008	60
NiPMAAm	0	100	1	0.9873	0.0000	0.013	54
	2.5	97.5	1	0.8097	0.1796	0.011	50
	5	95	1	0.6808	0.3099	0.009	51
	7.5	92.5	1	0.5830	0.4089	0.008	54
	10	90	1	0.5062	0.4865	0.007	55
DEGEEA	0	100	1	0.9914	0.0000	0.009	80
	2.5	97.5	1	0.8630	0.1293	0.008	78
	5	95	1	0.7594	0.2336	0.007	81
	7.5	92.5	1	0.6742	0.3195	0.006	76
	10	90	1	0.6028	0.3914	0.006	75
$mPEGMA_{300}$	0	100	1	0.9946	0.0000	0.005	75
	2.5	97.5	1	0.9094	0.0855	0.005	79
	5	95	1	0.8342	0.1610	0.005	78
	7.5	92.5	1	0.7673	0.2281	0.005	80
	10	90	1	0.7075	0.2882	0.004	75

Table S2. The molar $mPEGMA_{1100}$ comonomer content and the number average molecular weight, dispersity (*Đ*), cloud point (T_{CP}) and clearing point (T_{CL}), and the extent of hysteresis ($H = T_{CP} - T_{CL}$) values of the homopolymers and the $mPEGMA_{1100}$-based copolymers.

main comonomer	$X_{mPEGMA1100}$ (mol%)		M_n	*Đ*	T_{CP}	T_{CL}	*H*
	feed	NMR	(g/mol)		(°C)		
DEGEEA	0	-	2780	1.66	16.8	16.3	0.5
	2.5	2.1	3210	1.67	28.5	27.7	0.8
	5	7.5	3250	1.67	57.8	57.1	0.7
	7.5	9.9	3820	1.74	66.1	65.1	1.0
	10	11.7	3870	1.75	71.2	70.3	0.9
NiPAAm	0	-	3320	2.41	34.6	33.4	1.2
	2.5	1.8	4400	2.27	41.5	40.9	0.6
	5	5.3	5450	2.49	55.1	53.8	1.3
	7.5	11.3	4740	2.76	75.1	74.4	0.7
	10	12.7	4580	2.73	77.5	76.9	0.6
NiPMAAm	0	-	4050	1.87	50.6	48.9	1.7
	2.5	3.3	5640	1.9	59.5	58.2	1.3
	5	7	4890	1.94	68.6	68	0.6
	7.5	9.5	7420	2.02	74.6	74	0.6
	10	13.2	9080	1.86	82.6	81.9	0.7
$mPEGMA_{300}$	0	-	11240	3.57	69.5	68.6	0.9
	2.5	2.3	20200	2.17	72.6	71.7	0.9
	5	4.2	21550	2.07	75.2	74.8	0.4
	7.5	7.8	15900	2.48	80.5	79.6	0.9
	10	10.3	15250	2.77	82.7	81.9	0.8

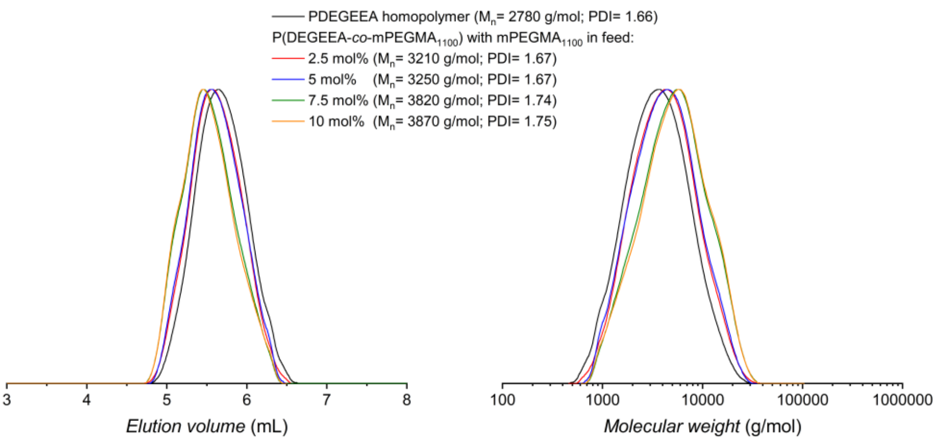

Figure S1. The GPC chromatograms and the molecular weight distribution curves of the PDEGEEA homopolymer and P(DEGEEA-*co*-mPEGMA$_{1100}$) copolymers.

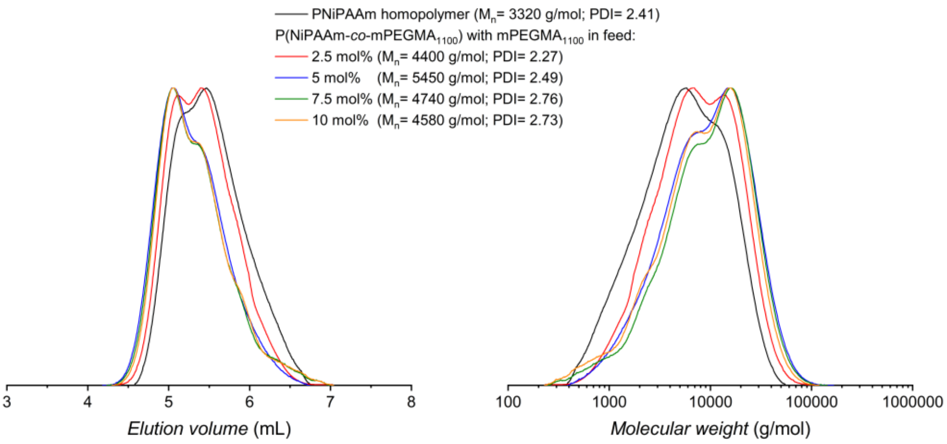

Figure S2. The GPC chromatograms and the molecular weight distribution curves of the PNiPAAm homopolymer and P(NiPAAm-*co*-mPEGMA$_{1100}$) copolymers.

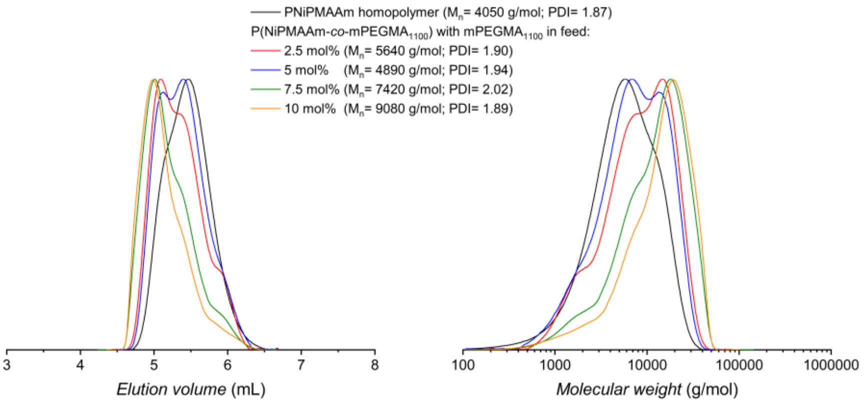

Figure S3. The GPC chromatograms and the molecular weight distribution curves of the PNiPMAAm homopolymer and P(NiPMAAm-*co*-mPEGMA$_{1100}$) copolymers.

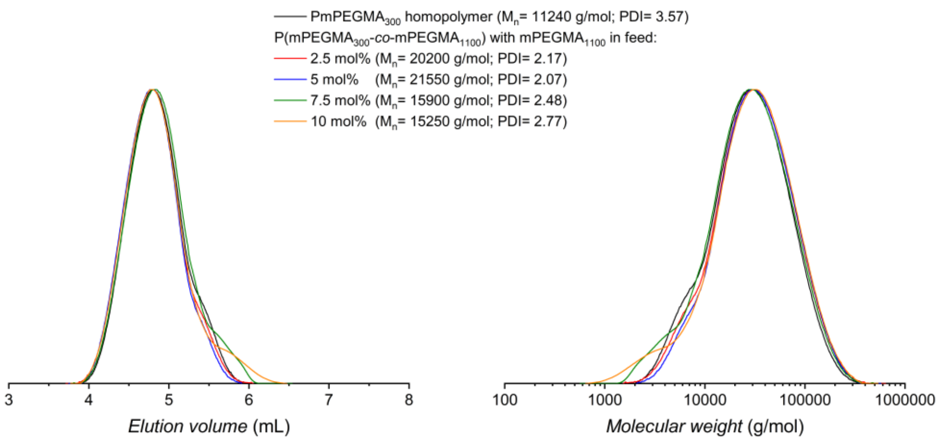

Figure S4. The GPC chromatograms and the molecular weight distribution curves of the $PmPEGMA_{300}$ homopolymer and P($mPEGMA_{300}$-*co*-$mPEGMA_{1100}$) copolymers.

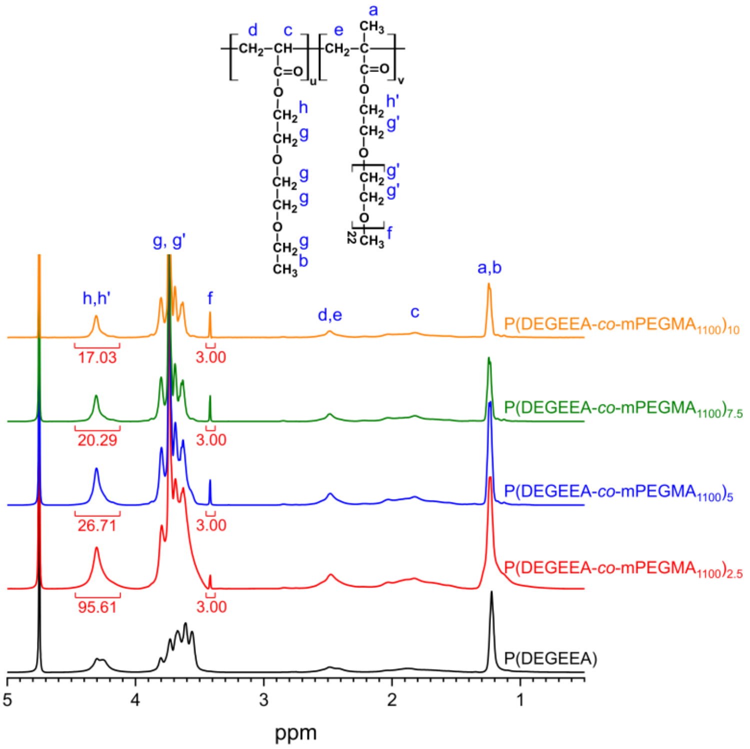

Figure S5. The ^{1}H NMR spectra of the PDEGEEA homopolymer and P(DEGEEA-*co*-$mPEGMA_{1100}$) copolymers (500 MHz, D_2O).

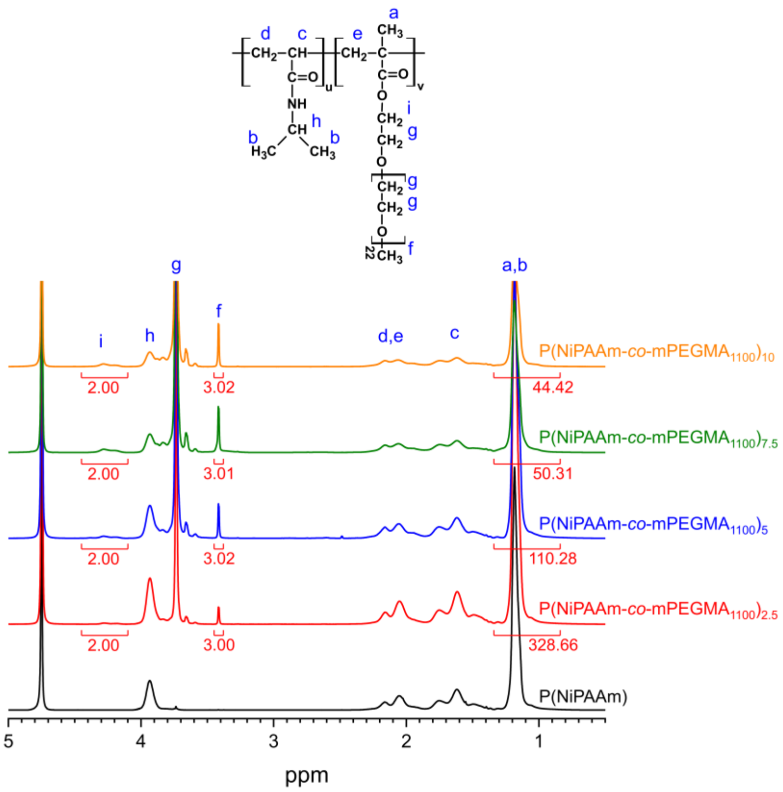

Figure S6. The ^{1}H NMR spectra of the PNiPAAm homopolymer and P(NiPAAm-*co*-mPEGMA$_{1100}$) copolymers (500 MHz, D_2O).

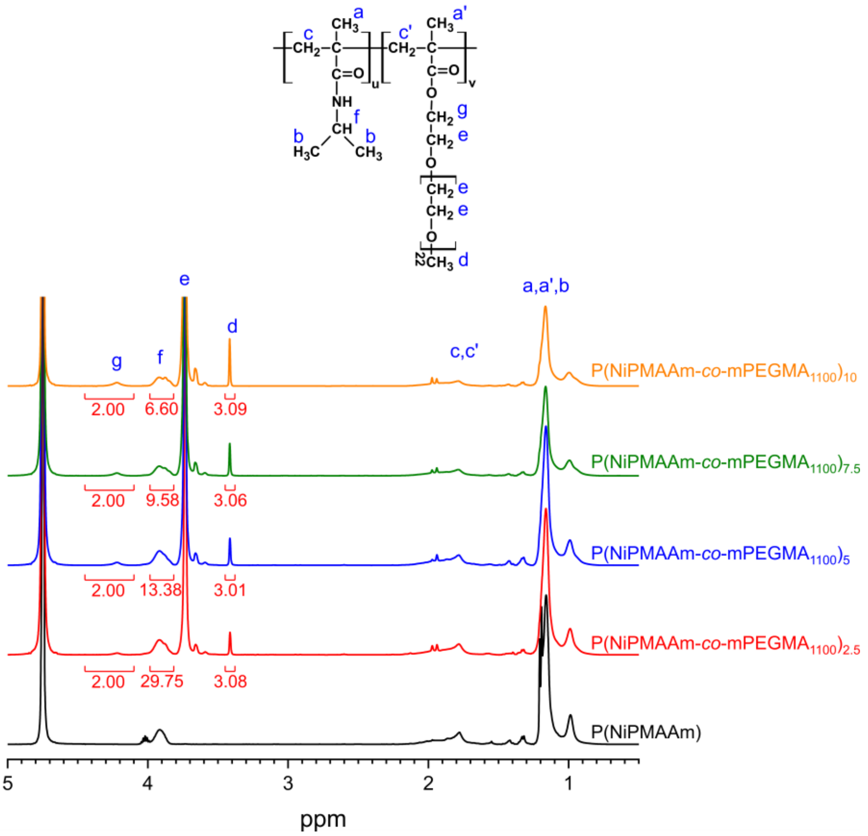

Figure S7. The ^{1}H NMR spectra of the PNiPMAAm homopolymer and P(NiPMAAm-*co*-mPEGMA$_{1100}$) copolymers (500 MHz, D_2O).

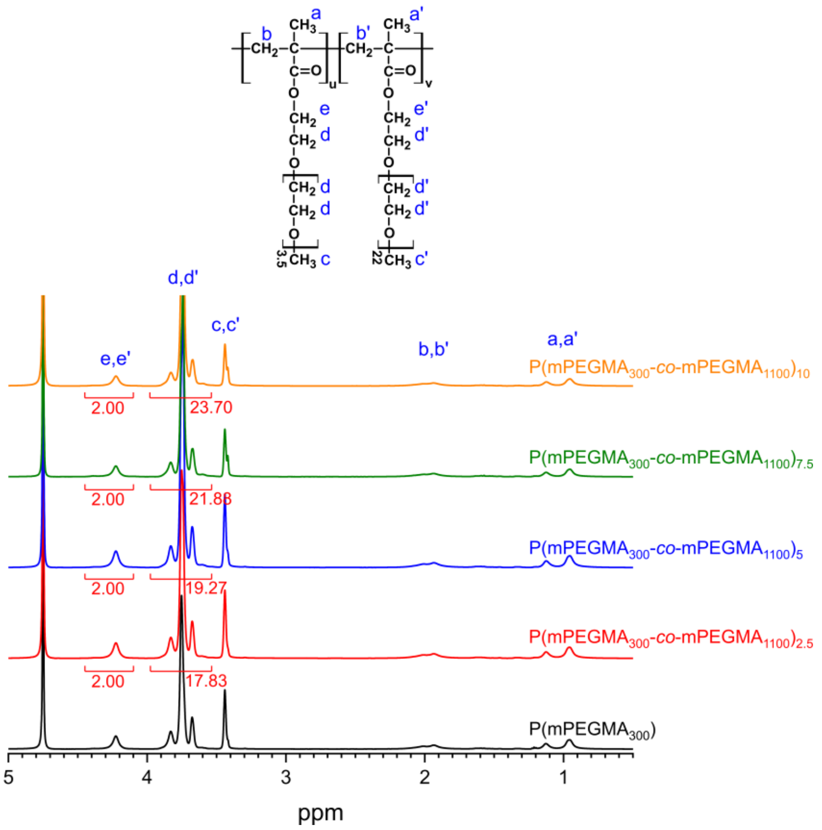

Figure S8. The ^{1}H NMR spectra of the $PmPEGMA_{300}$ homopolymer and P($mPEGMA_{300}$-*co*-$mPEGMA_{1100}$) copolymers (500 MHz, D_2O).

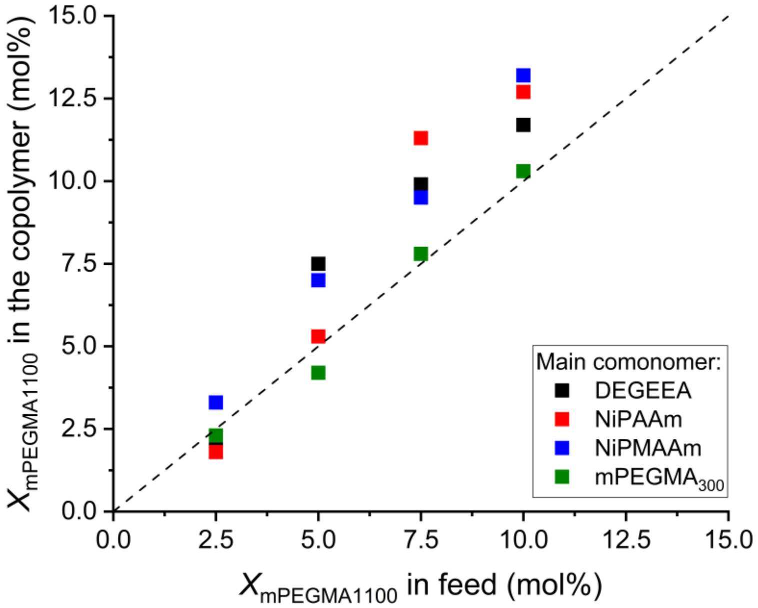

Figure S9. The $mPEGMA_{1100}$ content in the copolymers versus the molar fraction of the $mPEGMA_{1100}$ comonomer in the feed.

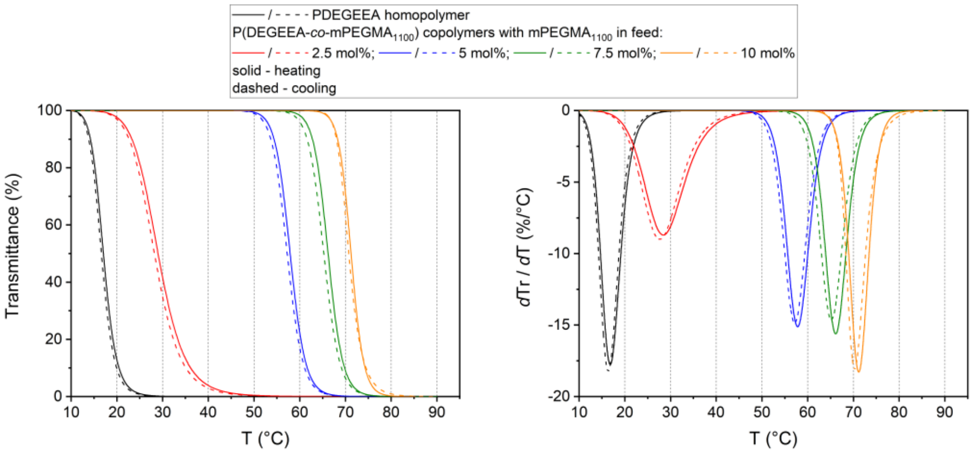

Figure S10. Transmittance–temperature curves (*left*) and their derivatives (*right*) during heating (*solid lines*) and cooling (*dashed lines*) of the PDEGEEA homopolymer and P(DEGEEA-*co*-mPEGMA1100) copolymers.

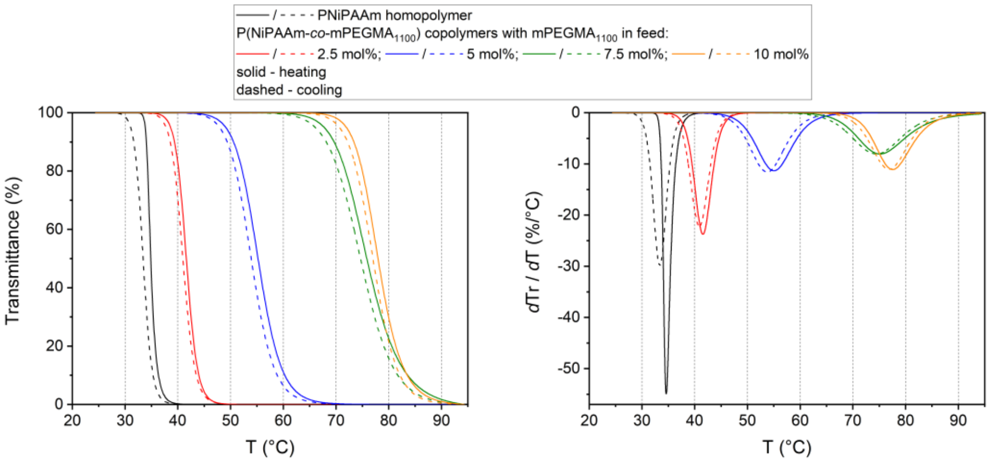

Figure S11. Transmittance–temperature curves (*left*) and their derivatives (*right*) during heating (*solid lines*) and cooling (*dashed lines*) of the PNiPAAm homopolymer and P(NiPAAm-*co*-mPEGMA1100) copolymers.

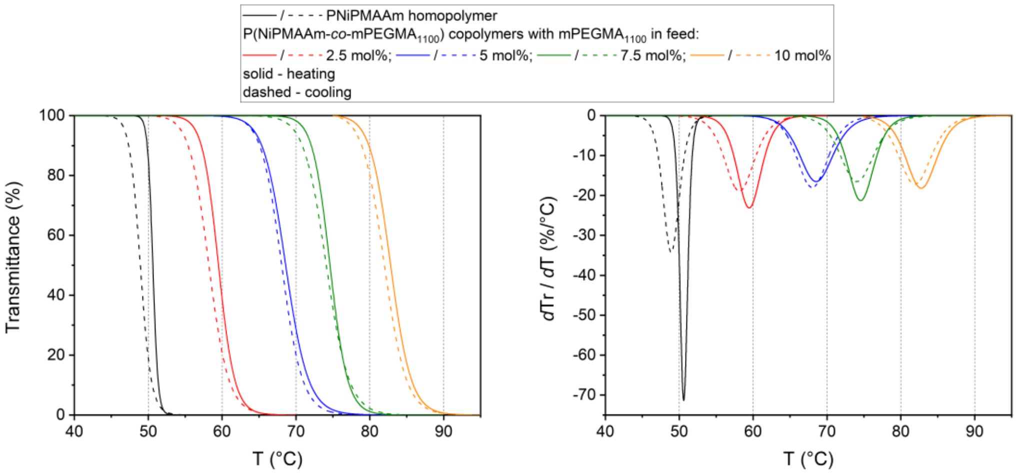

Figure S12. Transmittance–temperature curves (*left*) and their derivatives (*right*) during heating (*solid lines*) and cooling (*dashed lines*) of the PNiPMAAm homopolymer and P(NiPMAAm-*co*-mPEGMA1100) copolymers.

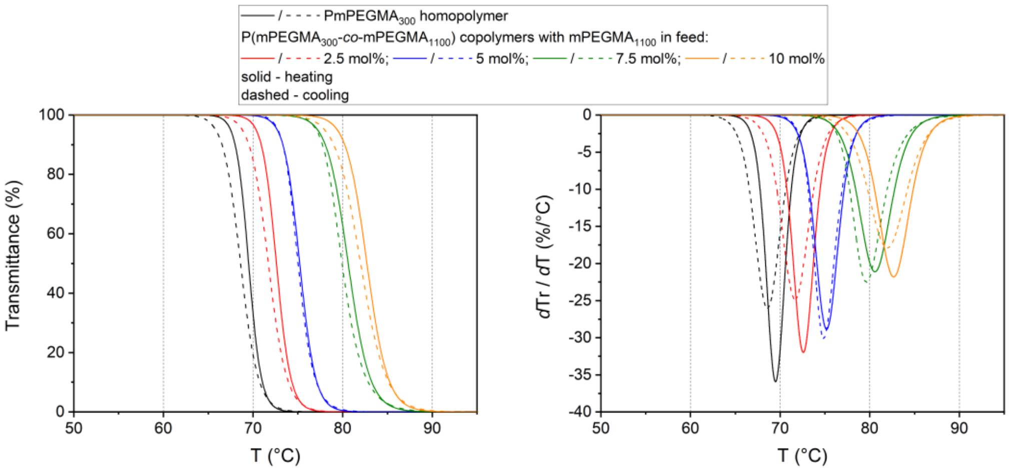

Figure S13. Transmittance–temperature curves (*left*) and their derivatives (*right*) during heating (*solid lines*) and cooling (*dashed lines*) of the PmPEGMA$_{300}$ homopolymer and P(mPEGMA$_{300}$-*co*-mPEGMA$_{1100}$) copolymers.

For Figures S14–S16 see the additional Supplementary File.

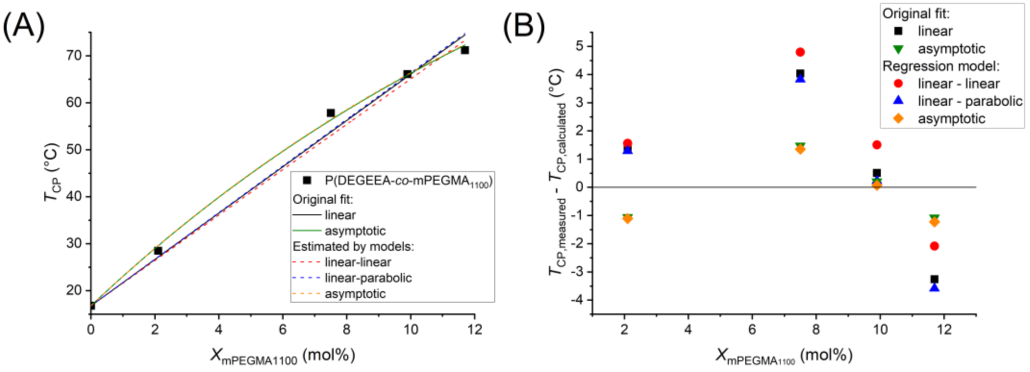

Figure S17. The cloud point temperature (T_{CP}) of the P(DEGEEA-*co*-mPEGMA$_{1100}$) copolymers as a function of the molar fraction of the mPEGMA$_{1100}$ comonomer with the original fitted linear and asymptotic functions and the estimated T_{CP} – $X_{mPEGMA1100}$ relationships using different models (A). The residual plot, i.e., the difference between the measured and the fitted and the calculated T_{CP} values, as a function of the composition (B).

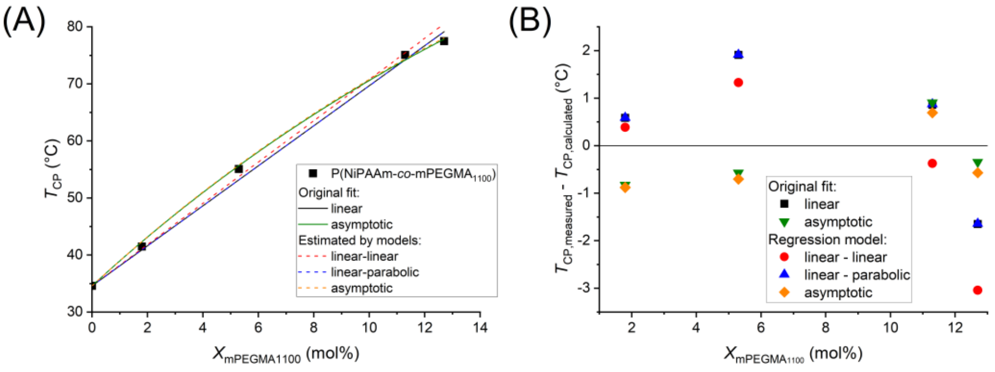

Figure S18. The cloud point temperature (T_{CP}) of the P(NiPAAm-*co*-mPEGMA$_{1100}$) copolymers as a function of the molar fraction of the mPEGMA$_{1100}$ comonomer with the original fitted linear and asymptotic functions and the estimated T_{CP} – $X_{mPEGMA1100}$ relationships using different models (A). The residual plot, i.e., the difference between the measured and the fitted and the calculated T_{CP} values, as a function of the composition (B).

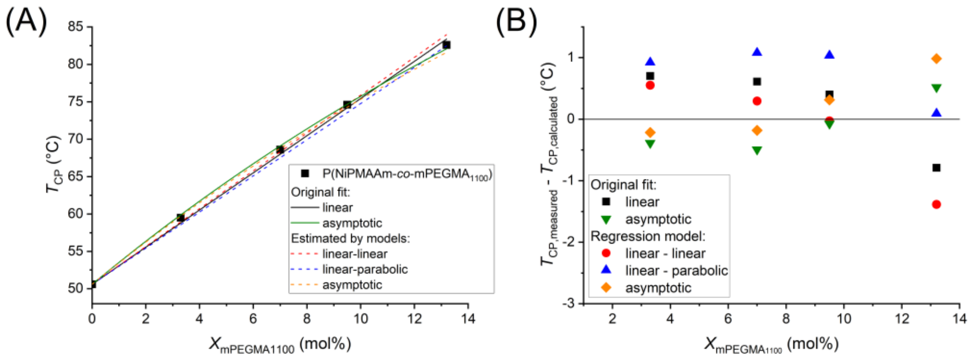

Figure S19. The cloud point temperature (T_{CP}) of the P(NiPMAAm-*co*-mPEGMA$_{1100}$) copolymers as a function of the molar fraction of the mPEGMA$_{1100}$ comonomer with the original fitted linear and asymptotic functions and the estimated T_{CP} – $X_{mPEGMA1100}$ relationships using different models (A). The residual plot, i.e., the difference between the measured and the fitted and the calculated T_{CP} values, as a function of the composition (B).

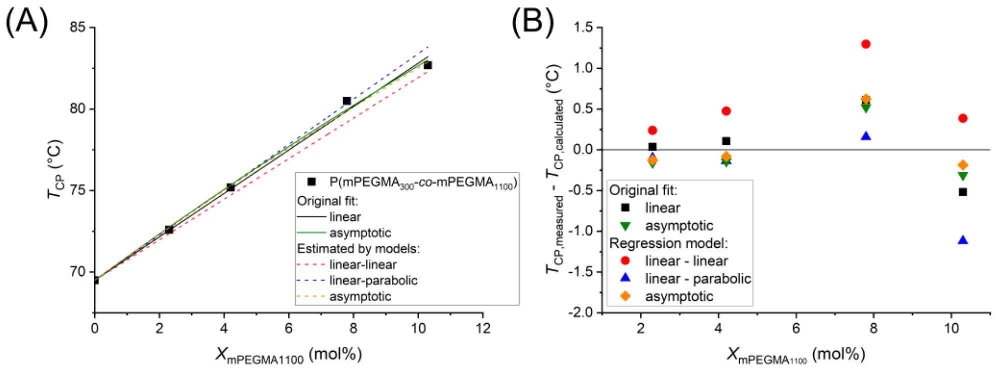

Figure S20. The cloud point temperature (T_{CP}) of the P(mPEGMA$_{300}$-*co*-mPEGMA$_{1100}$) copolymers as a function of the molar fraction of the mPEGMA$_{1100}$ comonomer with the original fitted linear and asymptotic functions and the estimated T_{CP} – $X_{mPEGMA1100}$ relationships using different models (A). The residual plot, i.e., the difference between the measured and the fitted and the calculated T_{CP} values, as a function of the composition (B).

Table S3. The measured and the estimated T_{CP} values of the $mPEGMA_{1100}$-based copolymers were obtained via the original fitted functions and using the developed estimation models with the mean absolute residuals.

main comonomer	$X_{mPEGMA1100}$ (mol%)	T_{CP} (°C)						mean absolute residuals (°C)		
			Fitted		Model			Model		
		Measured	Linear	Asymptotic	Linear	Parabolic	Asymptotic	Linear	Parabolic	Asymptotic
DEGEEA	2.1	28.5	27.1	29.6	26.9	27.2	29.6	2.79±1.75	2.55±2.01	0.88±0.71
	7.5	57.8	53.7	56.3	53.0	54.0	56.4			
	9.9	66.1	65.5	65.9	64.6	65.9	66.0			
	11.7	71.2	74.4	72.3	73.3	74.8	72.4			
NiPAAm	1.8	41.5	40.9	42.3	41.1	40.9	34.6	1.28±1.25	1.25±0.63	0.71±0.13
	5.3	55.1	53.2	55.7	53.8	53.2	42.4			
	11.3	75.1	74.3	74.2	75.5	74.2	55.8			
	12.7	77.5	79.2	77.8	80.5	79.1	74.4			
NiPMAAm	3.3	59.5	58.8	59.9	58.9	58.6	59.7	0.57±0.59	0.78±0.46	0.42±0.38
	7	68.6	68.0	69.1	68.3	67.5	68.8			
	9.5	74.6	74.2	74.7	74.6	73.6	74.3			
	13.2	82.6	83.3	82.1	84.0	82.5	81.6			
$mPEGMA_{300}$	2.3	72.6	72.6	72.8	72.4	72.7	72.7	0.60±0.48	0.38±0.49	0.25±0.25
	4.2	75.2	75.1	75.3	74.7	75.3	75.3			
	7.8	80.5	79.9	80.0	79.2	80.3	79.9			
	10.3	82.7	83.2	83.0	82.3	83.8	82.9			
