# Supplementary material for: When a Small Amount of Comonomer Is Enough: Tailoring the Critical Solution Temperature of LCST-Type Thermoresponsive Random Copolymers by PEG Methyl Ether Methacrylate with 1100 g/mol Molecular Weight
Source: Materials (Basel). 2025 Jan 15;18(2):372. doi: 10.3390/ma18020372 (PMC11766903; doi:10.3390/ma18020372)
Supplement: Supplementary file 1 [file materials-18-00372-s001.zip › materials-3412885-supplementary_Figures_S14-S16.pptx]

## Slide 1
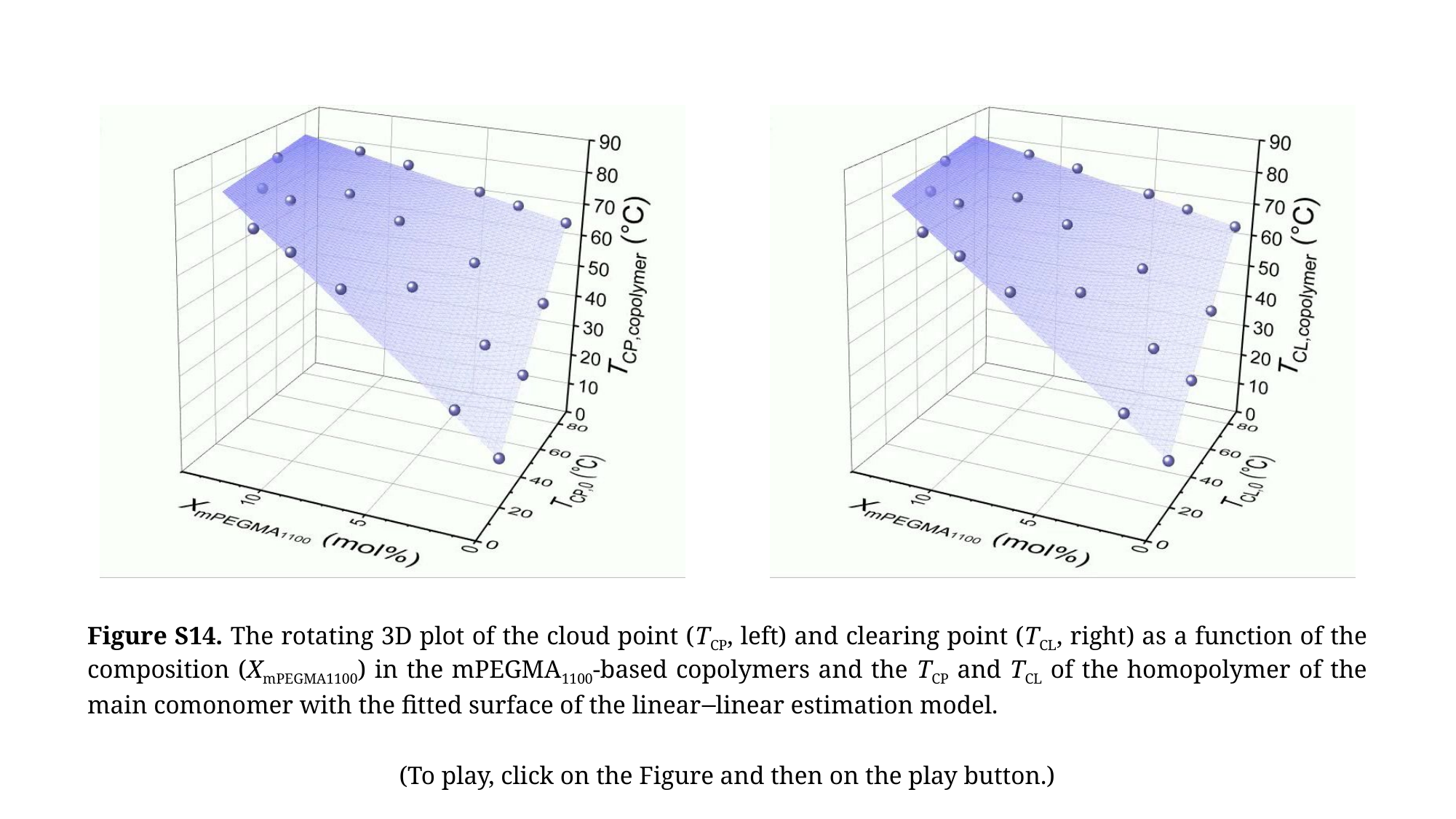

Figure S14. The rotating 3D plot of the cloud point (TCP, left) and clearing point (TCL, right) as a function of the composition (XmPEGMA1100) in the mPEGMA1100-based copolymers and the TCP and TCL of the homopolymer of the main comonomer with the fitted surface of the linear–linear estimation model.
(To play, click on the Figure and then on the play button.)

## Slide 2
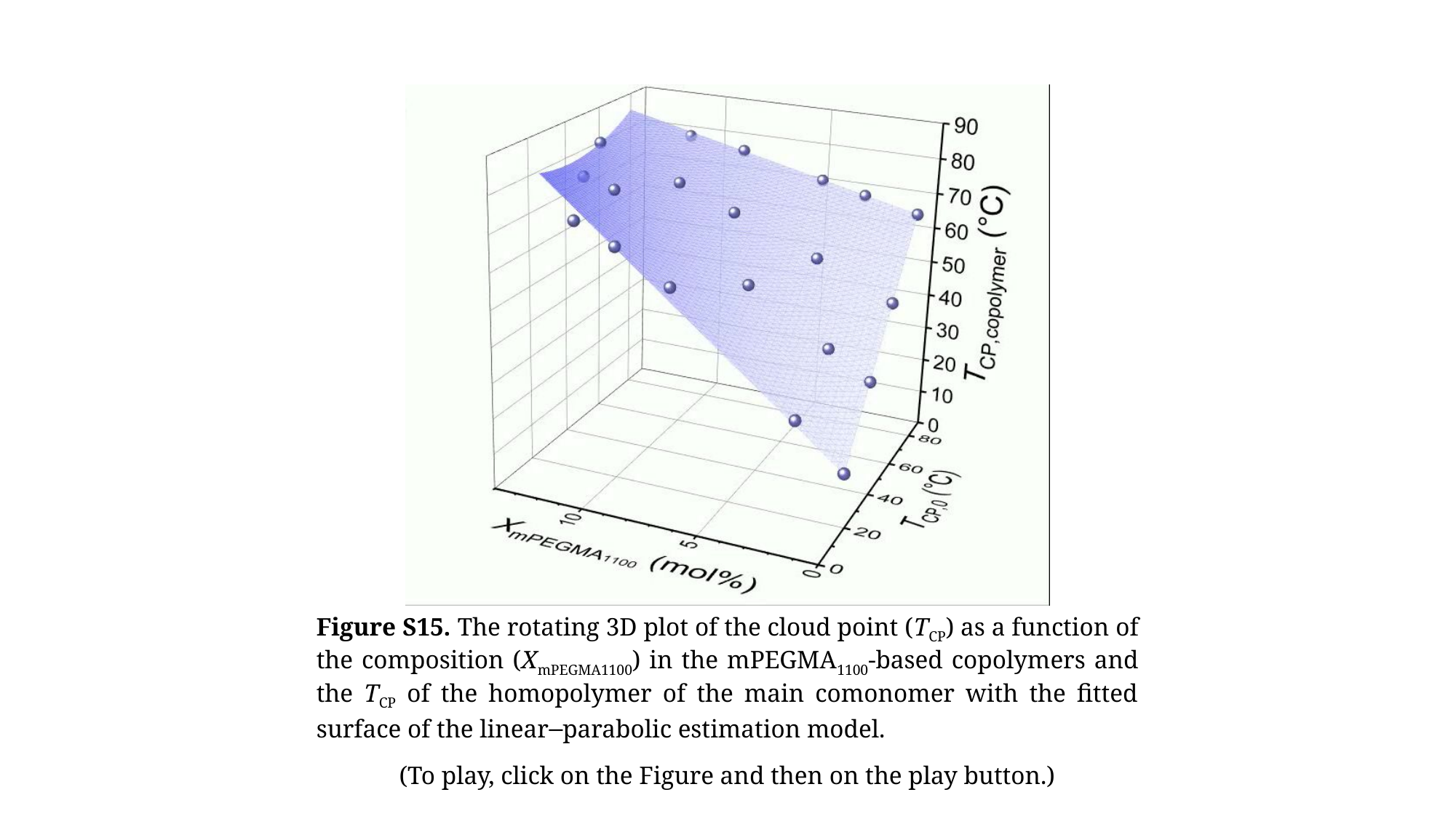

Figure S15. The rotating 3D plot of the cloud point (TCP) as a function of the composition (XmPEGMA1100) in the mPEGMA1100-based copolymers and the TCP of the homopolymer of the main comonomer with the fitted surface of the linear–parabolic estimation model.
(To play, click on the Figure and then on the play button.)

## Slide 3
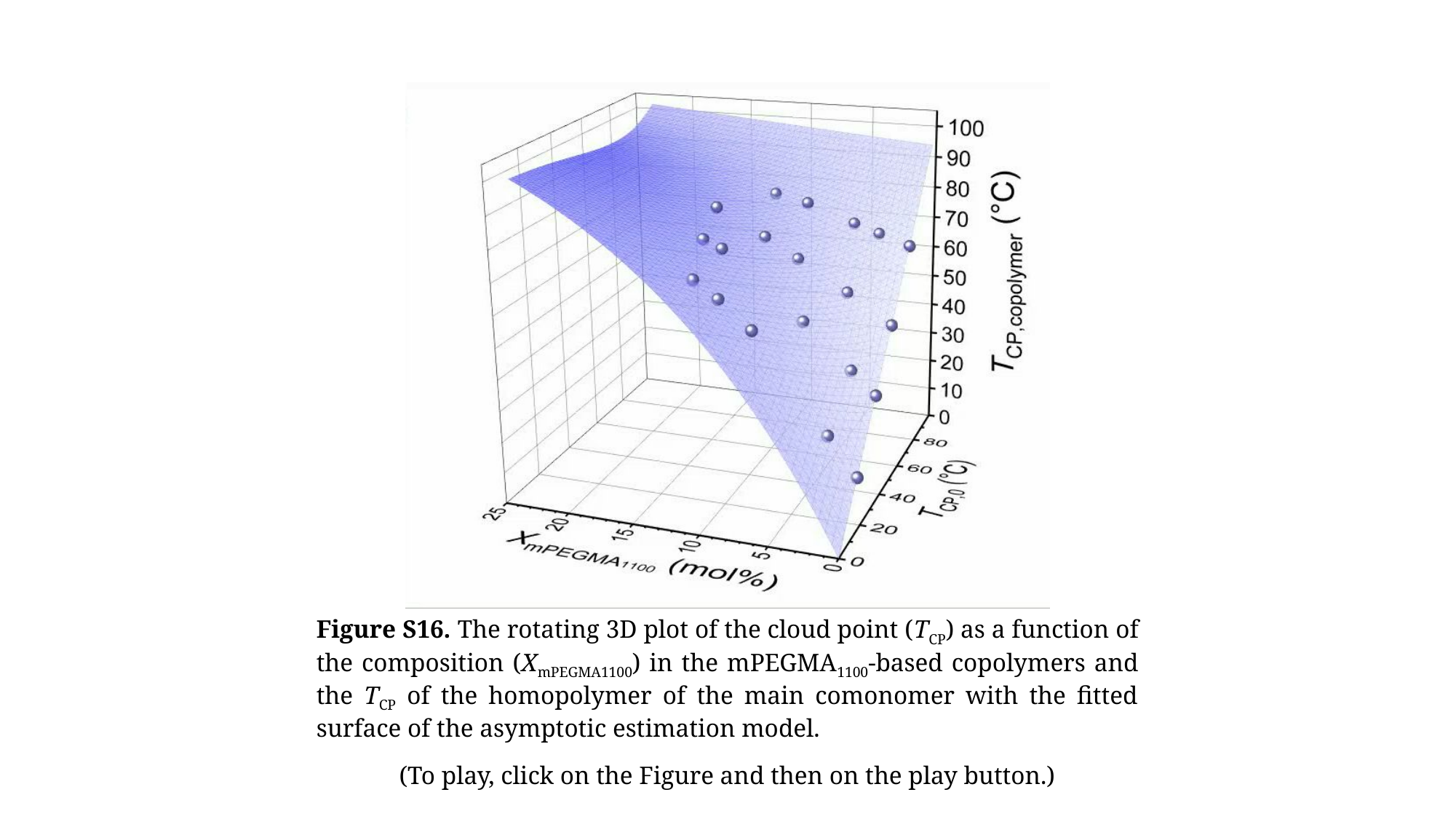

Figure S16. The rotating 3D plot of the cloud point (TCP) as a function of the composition (XmPEGMA1100) in the mPEGMA1100-based copolymers and the TCP of the homopolymer of the main comonomer with the fitted surface of the asymptotic estimation model.
(To play, click on the Figure and then on the play button.)
